# Spiritual care for Latinx youth with type 1 diabetes (T1D) and their caregivers: qualitative analysis of focus groups

**DOI:** 10.1186/s13098-026-02204-1

**Published:** 2026-06-10

**Authors:** Megan Visser, Jaquelin Flores Garcia, Rebecca Barber, Mark W. Reid, Stephanie S. Crossen, Jenise C. Wong, Jill Weissberg-Benchell, Diana Arellano, Alejandra Torres Sanchez, Frances Gonzalez, Salvador A. Lopez, Marysol Gonzales Granados, Jennifer K. Raymond

**Affiliations:** 1 The Center for Endocrinology, Diabetes and Metabolism, Children’s Hospital Los Angeles, Los Angeles, CA USA; 2 Institute for Nursing and Interprofessional Research, Keck School of Medicine, Children’s Hospital Los Angeles, University of Southern California, Los Angeles, Los Angeles, California, CA USA; 3 The Vision Center, Division of Ophthalmology, Department of Surgery, Children’s Hospital Los Angeles, Los Angeles, CA USA; 4 Department of Pediatrics, Division of Pediatric Endocrinology, University of California Davis School of Medicine, Davis, CA USA; 5 Department of Pediatrics, Division of Endocrinology, University of California, San Francisco, 550 16th Street, #4638, San Francisco, CA 94158 USA; 6 Ann and Robert H. Lurie Children’s Hospital of Chicago, Chicago, IL USA; 7 University of California, San Francisco, San Francisco, CA USA; 8 California University of Science and Medicine, Colton, CA USA; 9 University of California Davis, Davis, CA USA; 10 Keck School of Medicine, University of Southern California, Los Angeles, CA USA

**Keywords:** Type 1 Diabetes, Diabetes Technology, Latinx, Youth, Virtual Peer Support, Coping, Spiritual Care, Chaplain

## Abstract

**Background:**

Latinx youth with T1D experience systemic disparities in glycemic management and access to psychosocial support. Spiritual resources and needs may also impact diabetes distress and T1D management for youth and their caregivers. Professional chaplains are uniquely qualified to support patients and families with serious medical conditions; however, their services are not well understood and are underutilized in diabetes care. Our objectives included: (1) Identify spiritual experiences and needs of youth with T1D and their caregivers; and (2) Highlight opportunities for chaplains to provide spiritual care support to improve diabetes care.

**Methods:**

Qualitative analysis of spiritual care resources and needs reported in focus group sessions with Latinx youth (ages 13–17) with T1D and their caregivers. The framework method guided coding of transcripts and refinement of themes, which were interpreted based on professional spiritual care activities. Through constant comparison, a theory was developed to illustrate potential benefits of professional chaplain support in T1D.

**Results:**

With 13 youth and 13 caregivers, we identified themes that bear relevance for spiritual care support in diabetes, which included: loss of hope; negative self-image and shame; difficulty accepting reality of condition or wearing a diabetes device; grief; disconnection from community support; diabetes technology enhancing one’s life; and coping resources for spiritual well-being. These themes translate to practical benefits of a professional chaplain’s clinical activities. Spiritual care pathways for T1D care illustrate the intended effects, methods, and interventions of professional chaplains for improving resilience, quality of life, and support for self-management practices.

**Conclusion:**

Latinx youth and caregivers described deep emotional experiences related to living with T1D and self-management practices, which can be viewed and addressed through the lens of spiritual care. Our research is the first-of-its-kind to identify specific unmet needs in T1D care that could benefit from chaplaincy as part of the multidisciplinary team. Spiritual struggle related to managing T1D (e.g., isolation from others with T1D, negative self-image, grief) warrants specialized support. Further investigation should examine preferences of youth with T1D and their caregivers, specifically for spiritual care services as part of their T1D care, and integration of professional chaplain support in T1D care.

## Background

Approximately 2.1 million people in the United States have type 1 diabetes (T1D), including 314,000 youth (ages < 20); the highest annual increases have been seen in marginalized communities (e.g., Latinx, Asian or Pacific Islanders, Non-Latinx Black) [1, 2]. Historically excluded, racially and ethnically diverse populations with T1D are at higher risk of experiencing multilevel health disparities stemming from clinician bias, challenges accessing care, and a disproportionate burden of negative social drivers of health (e.g., food insecurity) [3]. Furthermore, Latinx youth with T1D have worse diabetes care outcomes, including higher hemoglobin A1c (HbA1c) levels, higher rates of psychosocial challenges, and lower use of diabetes technology compared to their white peers with T1D [4, 7]. For Latinx youth with T1D and their families, cultural factors such as religion and spirituality may impact their diabetes care management and outcomes [8, 9].

Among Latinx families coping with chronic conditions, spirituality is both a health resource and an important facet of their daily lives [8, 12]. Research indicates that positive spiritual coping supports the well-being of patients with serious chronic conditions, including diabetes [11]. A 2020 exploration of youth coping with diabetes found that Latinx youth and their caregivers identified the positive influence of integrating their spiritual practices with management of their illness. [12] Previous research on pediatric care for newly immigrated families highlights the importance of culturally responsive social and spiritual support [8]. Spiritual care focuses on fundamental needs connected to health and well-being, such as finding meaning and purpose in life, belonging and connection to others [9, 13]. Professional chaplains build rapport and relationship with patients and their families, in which a patient’s story, values and needs can unfold through deep reflective listening [14]. Professional chaplains develop spiritual care plans with specific outcomes and communicate with the multidisciplinary team [15]. Addressing spiritual care needs may be related or unrelated to religious identity, religious practice or specific faith communities [16]. As whole-person care in the US increasingly takes place in outpatient settings, chaplaincy research has highlighted the need for models (e.g., in-person, online) of spiritual care centered around outpatient clinic spaces [17, 18]. Spiritual care services also lag behind other disciplines in adopting technology resources, such as models of online peer support groups [19]. We must better understand the experiences of youth with T1D and their caregivers relevant to spiritual care [20, 22].

**Fig. 1:**
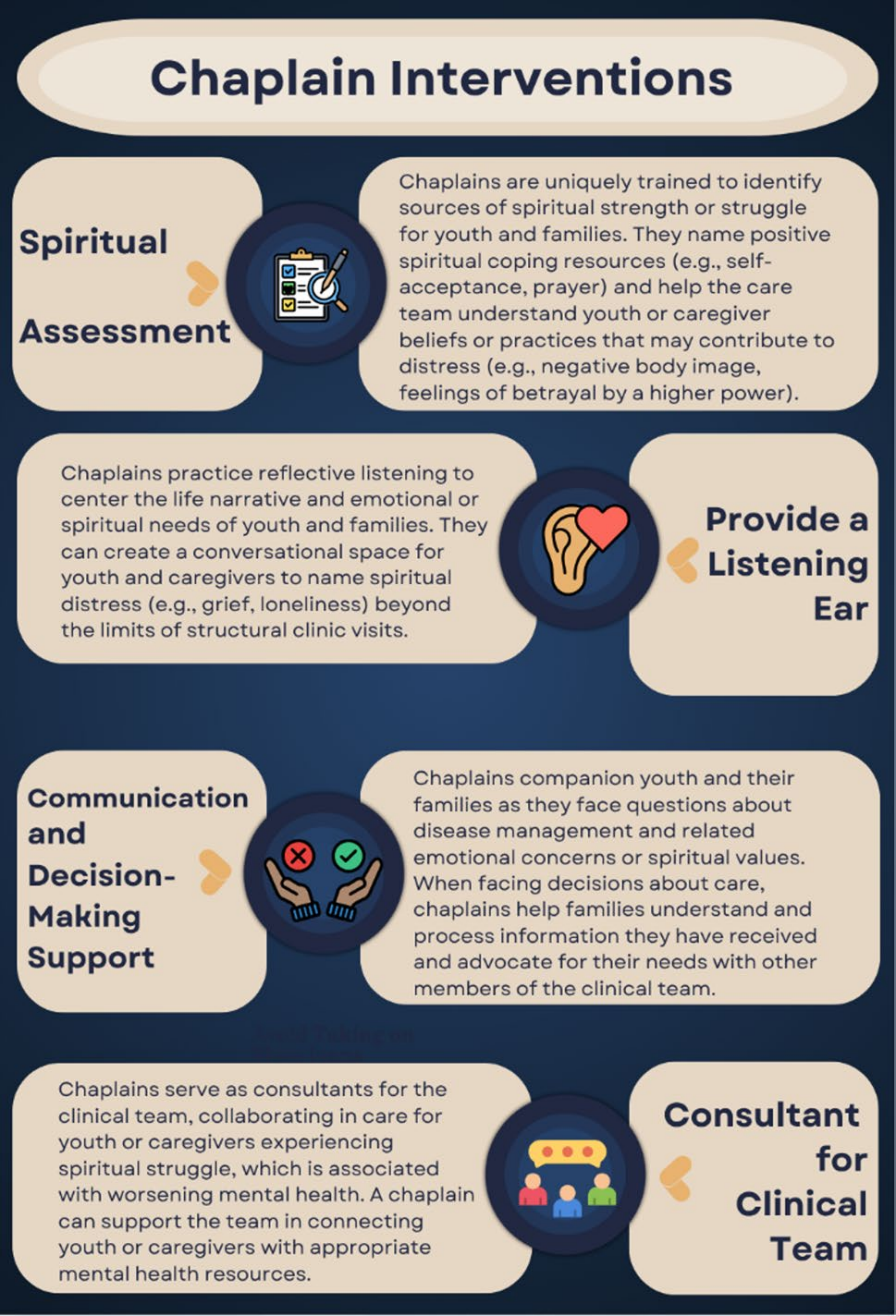
Chaplain Interventions

Spiritual care services offered by professional chaplains support patients and caregivers dealing with serious medical conditions by facilitating use of individuals’ spiritual beliefs, practices, or community support, and encouraging healthy communication and decision-making [23]. Chaplains are specialists uniquely trained to perform spiritual assessment and offer evidence-based interventions to address spiritual care needs [24, 27] (Fig. 1).

Chaplains also serve as consultants for the medical team and collaborators in treating patients experiencing spiritual struggle, which is associated with worsening symptoms of depression and other mental health conditions [27]. There is a well-documented gap between those families who need or desire chaplain services and those with access to a chaplain [28]. Also, chaplaincy is not well understood by the diabetes care and research community. [29] There is a need to identify spiritual needs and to highlight the opportunities for chaplains to provide spiritual care support to improve diabetes care, especially for marginalized communities [21, 30].

Latinx families and the approaches they use to care for their youth with diabetes are influenced by their unique lived experiences and cultural beliefs/values (e.g., familismo, fatalism, machismo, susto, curanderismo, fe en dios) [12, 21, 22]. This area of psychosocial care has not been extensively studied in this population, and there is a critical need to understand and provide culturally tailored care for Latinx families living with diabetes. This study aims to: (1) Identify spiritual experiences and needs of youth with T1D and their caregivers; and (2) Highlight opportunities for chaplains to provide spiritual care support to improve diabetes care.

## Methods

The present study used data collected from Latinx youth with T1D and their caregivers who completed a focus group or interview for the first phase of the DREAM (Device use Reimagined through Education And Mentorship) study, which was conducted in 2023 at three pediatric diabetes centers in California (Children’s Hospital Los Angeles [CHLA], University of California Davis [UCD] and University of California San Francisco [UCSF]). Latinx youth (ages 13–17) with T1D and their caregivers were recruited to participate in DREAM focus groups if they spoke English and/or Spanish. Focus groups were conducted on a videoconferencing platform (e.g., Zoom, Teams), with up to six participants each and lasted approximately 1 h to 1.5 h. Research team members (e.g., a child psychologist, bilingual nurse/diabetes educator) conducted each group in either English or Spanish, using a discussion guide that consisted of questions about the use of diabetes technology and interests in virtual peer groups. All focus groups were recorded, transcribed, and if needed translated to English. Each participant received a $50 gift card. [31].

### Analysis

All of the Latinx youth with T1D and caregivers who participated in DREAM Phase 1 focus groups were included in the current qualitative analysis of spiritual resources and needs. Informed by grounded theory principles, we used the Framework method [32] to analyze the focus group data and explore the potential benefits of professional support for spiritual coping. The analysis team was comprised of a postdoctoral research fellow (who is a sociologist and hospital chaplain) and a senior medical writer with extensive diabetes research experience in marginalized communities from the original DREAM research team. First, the verbatim transcripts of the focus groups were reviewed by the analysis team. Individually, team members used deductive and inductive coding methods to develop an initial codebook based on the first few transcripts, using Dedoose qualitative software [33]. The analysis team met to compare individually coded sections of transcript and group codes into categories. The codes were defined to create a working analytical framework. The analytical framework was applied to the subsequent transcripts using the existing categories and codes in Dedoose. A spreadsheet was created to manage and summarize data in a framework matrix. The analysis team met to discuss emerging themes and further interpret the data by mapping connections between the data and pre-existing constructs in professional health care chaplaincy. During meetings, the team reflected on potential biases and fidelity to the participants’ voices in our analytical process. Professional chaplain spiritual care activities were used as an interpretive lens to explore the meaning of these themes for interdisciplinary T1D care. Using a constant comparative technique, emerging themes and interpretations were refined during analysis meetings. A theory was developed to illustrate potential benefits of professional chaplain support in T1D care [23]. 

### Trustworthiness and authenticity

To enhance the trustworthiness of our findings, the analysis team used several strategies. Prolonged engagement with the data (18 months) and the inclusion of numerous participant quotations supports the credibility of the study. We addressed dependability by maintaining documentation of codebook versions and theme development over time. Confirmability was strengthened by including a senior medical writer with expertise in diabetes research who was not a chaplain nor another clinician on the analysis team. The team attended to each researcher’s positionality to ensure that coding of the data was grounded in the participants’ voices. We facilitated transferability by describing the research context of DREAM and the characteristics of our participants, which will allow readers to assess if our results are relevant for other diabetes care settings. It is important to acknowledge the interpretive role of the researchers in applying insights from chaplain interventions to the lived experiences of the focus group participants. The analysis team interpreted participants’ quotations as they bear relevance for chaplain interventions, developing spiritual care pathways as a device to illustrate potential benefits of chaplain availability in T1D care. Spiritual care services focus on listening deeply to fundamental needs related to health and well-being versus tending to formal religious identity or tradition [9, 13]. Reinforced by this philosophy, our research team sought to genuinely represent participant’s perspectives and fairly interpret their meanings in the analytical process.

## Results

A total of 13 youth and 13 of their family members participated in 11 focus groups (Table 1). 46% of youth identified as having female gender. Youth identified their race as Other (46%) or White (38%) and two youth declined to state their racial background. All participants identified their ethnicity as Hispanic or Latino/a/x. All youth were primarily English speaking and 69% of caregivers were primarily Spanish speaking. Most youth (77%) were bilingual in Spanish and English. 77% of youth were publicly insured. At the time of the study, youth had T1D for an average of 5.5 years. Diabetes technology was used by most in the cohort, with 85% (11) having ever used CGM and 69% (9) having ever used an insulin pump. Family members reported a median household income of $35,000–49,000 (USD) and more than half (62%) reported completing some high school or more education. Most youth (92%) and family members (85%) reported being very comfortable with using technology, such as laptops, computers, tablets, or cell phones.

**Table 1:** Youth and Family Demographics

Characteristics Mean (SD) or *n* (%)	Total	
	Youth (*N* = 13)	Caregiver (*N* = 13)
**Age (years)**	14.46 (1.27)	
**Race** Pacific Islander White Other Decline to State	0 5 (38%) 6 (46%) 2 (16%)	3 (23%) 2 (15%) 7 (54%) 1 (8%)
**Ethnicity** Hispanic or Latino/a/x	13 (100%)	13 (100%)
**Gender** Female Male Decline to State	6 (46%) 6 (46%) 1 (8%)	
**T1D duration (years)**	5.15 (1.99)	
**Language** Primarily Spanish Speaking Primarily English Speaking	0 13 (100%)	9 (69%) 4 (31%)
**Bilingual**	10 (77%)	5 (38%)
**HbA1c** *(n= 10)*	9.59 (2.38)	
**Insurance** Public Private Both Public and Private	10 (77%) 2 (15%) 1 (8%)	
**Comfort with general technology (e.g.**,** tablet**,** computer**,** phone) use** Very Comfortable Somewhat Comfortable Somewhat Uncomfortable Very Uncomfortable	12 (92%) 1 (8%) 0 0	11 (85%) 2 (15%) 0 0
**Diabetes Technology** CGM Use Insulin Pump Use	11 (85%) 9 (69%)	
**Education Completed** Some Elementary School Some Middle School Some High School High School Graduate/GED Trade/Vocational/Technical School Some College/Associates Degree Bachelors Degree Graduate Degree Other		2 (15%) 3 (23%) 3 (23%) 0 0 3 (23%) 0 1 (8%) 1 (8%)
**Household Income Brackets (USD)** <$25,000 $25,000–34,999 $35,000–49,999 $50,000–74,999 Not sure		2 (15%) 3 (23%) 4 (31%) 0 4 (31%)

Participants shared personal experiences that connect to our operating definition of spirituality: “the aspect of humanity that refers to the way individuals seek and express meaning and purpose, and the way they experience their connectedness to the moment, to self, to others, to nature and to the significant or sacred.” [34] Youth and caregiver participants reported social and emotional concerns that bear relevance for spiritual care: loss of hope, being disconnected from communities of support, difficulty accepting their T1D diagnosis or diabetes devices, grief; experiences of negative self-image or shame; diabetes technology enhancing life; and coping resources for spiritual well-being (Table 2).

**Table 2:** Participant Quotations

Themes	Quotation
**Loss of Hope**	*Overwhelmed with monitoring* “Helplessness of not being able to see the numbers” – caregiver *Wanting to give up* “There are times when you say, ‘I can’t do this anymore’ but you have to be strong so she can be well” – caregiver
**Disconnected from Community Support**	*Feeling isolated from family* “I had no family support, and the kids were in COVID. And so I had my two kids at home every single day…and they were home alone every day and I was at work. And then I had no family support to go check on them”- caregiver *Lack of adults with T1D knowledge* “No one do it with bad intentions, but it got to the point where I would say, you know what, thank you for your help – because they would come with vials of things. ‘Give this to your son. It’s going to cure his diabetes’”- caregiver *Youth isolated from peers* “She doesn’t want to use it [sensor] because she says that she feels that everyone is looking at her. She feels that everyone is looking at her, and she doesn’t want it. She doesn’t like when people find out she has diabetes” - caregiver
**Difficulty Accepting T1D Diagnosis or Diabetes Devices**	*Difficulty accepting diagnosis* “It was also difficult for her to accept her condition, and she still has difficulties in wanting to accept or take care of her condition.” – caregiver “Something that I feel would affect someone trying to join the [virtual peer] group is, they don’t accept who they are just yet. I don’t know how to explain it. They’re having a difficult time to embrace it. Because I know, at first, I had a difficult time” - youth *Youth opinion that diabetes is for older adults* “She was sad. ‘Oh, I don’t want to have diabetes, diabetes is for older people.’ I say ‘No mama, don’t say that, it’s something that happens in life. We don’t know why.’” – caregiver *Psychological burden of diagnosis* “For her it is like a psychological burden and something that she struggles to accept and be willing to take part in taking care of herself” - caregiver *Caregiver with difficulty accepting diagnosis* “There are times when this becomes very frustrating for me. I don’t want my daughter to have this illness. And there are times when you say, ‘I can’t do this anymore’ but you have to be strong” – caregiver *Difficulty accepting device* “I guess they see my pump and I just don’t like people staring at me and judging me.” – youth *Emotionally not ready for a device* “He [my son] feels he’s not ready yet. Because he says that with that whole process he has to be 100% sure of what it is and how reliable it is, whether it works or doesn’t work” – caregiver “As soon as she saw it, she got hysterical, ‘I don’t know why they sent this. I already told you that I am not going to use it or wear it’” - caregiver
**Grief**	*Loss of pre-diabetes daily life* “Before, my son would wake up and say good morning. Not anymore. The first thing I say to him is, ‘What are your levels like?’ Life changes for the whole family” – caregiver *Loss of perceived similarities to peers* “It’s living their lives completely different from other children” – caregiver *Grieving diagnosis* “There are times when this becomes very frustrating for me. I don’t want my daughter to have this illness” – caregiver
**Negative Self-Image**	*Previous bullying experiences* “I just don’t want it because I used to get bullied as a young kid because I had diabetes” – youth *Negative self-image increased with age* “It was easier before because she was younger, but now that she’s older, it’s harder because it’s not the same as before because she is with older children and they don’t think about what they are going to say and they make comments that make her feel bad, so she prefers to not share things” – caregiver *Not wanting to be seen* “If we had company at the house, she would say, ‘Mom, I don’t want anyone to look at me that I’m doing my things’” – caregiver *Not wanting to use an insulin pump* “Because I’ve seen it [the insulin pump] is visible a lot. It’s pretty much visible. That’s why” – youth “If I want to use one [a pump], I would have to see how they look-they’re chunky” – youth *Self-harm* “I don’t know if she feels like she’s going to be judged or they’re going to feel sorry for her. I don’t know, but she doesn’t like people to know anything about it. She doesn’t like it when they see her pump or find out she has diabetes…there was even a time when she was cutting herself” – caregiver
**Diabetes technology enhances life’s meaning**	*Freedom with devices* “He feels more normal having the technology. He doesn’t have to stop his life. He doesn’t have to think about what he’s eating to give himself a shot. Definitely more freedom and flexibility of what he can do and timing of what he can do” – caregiver *Flexibility at school* “He’s not having to carry around the sharps container and his insulins. And he doesn’t have to go to the health office at school” - caregiver *Improved family relationships* “it was a lot less battling over his health condition. He would eat. It was just great as a mom…More eating, less battling over giving insulin” – caregiver *Not a one-size-fits-all solution* “The pump helps him somewhat. But, well, he has to do more of his part. It is more of a psychological problem that has nothing to do with pump” – caregiver
**Coping Resources**	*Sources of community at diabetes conference* “The first two times that I took him [to a diabetes conference], it was a big change for him. He saw that there were many children who were using pumps, sensors, and they told him that it had changed their life using the sensor and the pump and he saw the kids with shorts and t-shirts showing their pumps, their sensors and that was how he decided to use it” – caregiver *Interest in getting to know others with diabetes* “In my school, there isn’t many people with diabetes and even if there is, I really don’t get to see them often, or talk to them, and aren’t really in my classes. So it would be nice to…get to know other people who have diabetes” – youth “I think just being there with other people that have experienced the things that you could have experienced is a good thing to know, knowing you’re not alone.” - youth *Caregiver respect for youth’s privacy* “We talked and I told her that she could go to her room and do it [administer insulin] and then come out” – caregiver *Clinical space for reflective listening* “It’s like making them proud of what they’re learning and the way they’re improving or the way they’re reflecting on their care” – caregiver *Educating other youth about T1D* “If anybody has questions, that I should be able to answer them, if I feel comfortable doing it” – youth *Shared appointments encourage youth community* “When we went to a hospital appointment with families, she saw there are more children, more young people and she kind of accepted it little by little” - caregiver

These themes interconnect with spiritual care pathways of chaplain activities, as described in the taxonomy of chaplain activities [23] (Table 3). Spiritual care pathways illustrate the intended effects, methods and interventions of professional chaplains. We further interpreted these pathways for T1D spiritual care in terms of improving resilience, quality of life, and support for self-management practices among youth with T1D and their caregivers.

**Table 3:** Spiritual Care Pathways for T1D

Themes	Spiritual Care Pathway (Effects – Methods – Intervention)
**Loss of Hope**	Promote a sense of peace - Explore hope - Communicate needs or concerns to others
**Disconnected from Community**	Lessen someone’s feelings of isolation - Identify supportive relationships - Facilitating groups
**Difficulty Accepting T1D Diagnosis or Diabetes Devices**	Meaning-making - Demonstrate acceptance - Ask questions to bring forth feelings
**Grief**	Help someone feel comforted - Journeying with someone in the grief process - Provide grief resources or processing session
**Negative Self-Image**	Preserve dignity and respect - Encourage self-care - Offer emotional support
**Diabetes Technology Enhances Life’s Meaning**	Aligning care plan with patient’s values - Ask guided questions about purpose - Perform a blessing
**Coping Resources**	Assist with identifying strengths - Facilitate communication - Discuss coping mechanisms

### Loss of hope

Multiple caregivers reported a loss of hope, specifically being overwhelmed with monitoring their children’s care and feelings of wanting to give up. When reflecting on their experience of loss of hope, caregivers drew on their experiences shortly after their children were diagnosed with T1D. Some caregivers described these feelings as “helplessness,” regarding managing their children’s diabetes before they had access to continuous glucose monitoring. One mother described giving up hope: “there are times when you say, ‘I can’t do this anymore’ but you have to be strong so she can be well.” Other caregivers noted a general loss of hope based on caregiving challenges as a single caregiver, financial concerns exacerbated during their children’s hospitalization, and a lack of peer support.

*Caregivers’ descriptions of their loss of hope illuminate a spiritual care pathway of chaplain activities: chaplains promote a sense of peace by exploring sources of hope and communicating needs or concerns to others.*

### Disconnected from community support

Youth and caregivers reported feeling disconnected from community support, such as being isolated from extended family, peers at school, or other families dealing with T1D. They expressed a need for community support and desire to share discussion space with other families of youth with T1D.

*Feeling isolated from family and peers.* Caregivers described social isolation from family members and youth’s peers at school. Youth and caregivers described the youth experiences of hiding diabetes technology from peers at school. One caregiver reported that her daughter sharing that she had T1D with her peers was an alienating experience, leading to depression and self-harm: “there was even a time when she was cutting herself.”

*Lack of other adults with T1D knowledge.* Caregivers reported that other caregivers in their social circles had many misconceptions about T1D that negatively affected their relationships with these adults. A Spanish-speaking caregiver said that other adults would try to give them items to “cure” their child’s diabetes. An English-speaking caregiver said that their extended family members were not interested in learning about their child’s diabetes.

*Desire for peer group engagement.* Most youth participants shared that they would like to be able to gather in groups with other youth with T1D for support, education, and community-building, especially in groups that meet separately from caregivers or adults. In an interview, an English-speaking caregiver shared that they wished family members could attend a group with them to learn about diabetes and their child’s care needs. In another interview, a Spanish-speaking caregiver remarked that having their child in a group with other youth with T1D would be a great benefit to their mental health.

*A spiritual care pathway of chaplain activities applies to these needs: chaplains lessen someone’s feelings of isolation for patients and families by identifying supportive relationships and facilitating groups.*

### Difficulty accepting diagnosis or device

Youth and caregivers reported challenges of youth to accept their T1D diagnosis or reluctance to adopt diabetes technology, such as an insulin pump or continuous glucose monitoring.

*Diagnosis.* Caregivers in the Spanish-speaking focus groups reported that their children had difficulty accepting their T1D diagnosis. One caregiver shared that their child believed that diabetes was only a disease for older adults: “She was sad. ‘Oh, I don’t want to have diabetes, diabetes is for older people.’ I say, ‘No mama, don’t say that, it’s something that happens in life. We don’t know why.’” Another caregiver reported their own difficulty accepting the condition. Some caregivers reported their children experienced anxiety and depression related to accepting their diabetes diagnosis. Some youth did not wish to administer insulin or participate in managing T1D. Most focus groups of Spanish-speaking caregivers reported that their children’s diagnosis was a source of stress for themselves, their child, and their families.

*Devices.* Caregivers described how their children did not feel “ready” or felt “afraid” to initiate use of diabetes technology. Another Spanish-speaking caregiver said that their son was “afraid” of starting to use technology and they would not initiate the use of any devices until he felt ready. According to a caregiver, a female youth was very resistant to accepting diabetes technology when presented with the option. Youth reported that the visibility of devices made them reluctant to initiate device use.

*Participants’ hesitancy to accept aspects of T1D or diabetes devices highlight a spiritual care pathway: chaplain interventions assist youth and caregivers with meaning-making by demonstrating acceptance and asking questions to bring forth one’s feelings.*

### Grief

Diagnosis with diabetes signaled a turning point in the self-identity of youth with T1D, in which youth experience loss and subsequent feelings of grief. One caregiver reported, “It’s living their lives completely different from other children.” Caregivers described the loss of the daily life that they had with their children prior to dealing with diabetes and a loss of their prior relationships with their children. Caregivers described their feelings of grief about their children’s diagnosis with T1D. Another caregiver reported anticipatory grief for their child, who shared with them that they felt their early death from diabetes was inevitable, based on an experience of grieving the loss of a relative with T1D.

*In the spiritual care pathway to address grief*,* chaplains journey with individuals in the grieving process*,* helping them to feel comforted and providing resources or grief processing sessions*.

### Negative self-image or shame

Spanish-speaking caregivers and English-speaking youth shared that youth had negative self-image and shame around their diagnosis or self-management practices, such as administering insulin or using diabetes technology. Experiences with negative self-image and shame led youth to restrict their eating or avoid use of diabetes technology. Youth reported negative experiences with body image or peer relationships related to having T1D, informed by earlier experiences with bullying at school. A Spanish-speaking caregiver reported that their daughter said, “Mom, I don’t want anyone to look at me when I’m doing my things.” Another Spanish-speaking caregiver shared that their child had engaged in self-harm: “She doesn’t like it when they see her pump or find out she has diabetes…there was even a time when she was cutting herself.”

*Participant voices illuminate a connection to a spiritual care pathway: chaplains preserve dignity and respect for individuals facing negative self-image or shame by encouraging self-care and offering emotional support.*

### Diabetes technology enhances life

Caregivers and youth shared how diabetes technology enhanced youth’s lives by improving their quality of life and offering them more “freedom” in their daily lives. A caregiver stated that technology offered their son more flexibility, especially at school: “he feels more normal having the technology. He doesn’t have to stop his life.” The caregiver reflected that diabetes technology improved their relationship with their child and reduced experiences of feeling overwhelmed. While caregivers frequently reflected that diabetes technology had improved their children’s lives, one Spanish-speaking caregiver shared that it was not a full solution to their child’s emotional distress, which was a barrier to managing their T1D well.

*Diabetes devices may be a spiritual resource for youth and caregivers that corresponds to a spiritual care pathway: chaplains seek to align care plans with patient values by asking guided questions about a treatment’s purpose and performing a blessing of medical technology.*

### Coping resources for spiritual well-being

Focus group participants identified several coping resources for dealing with negative self-image or shame regarding self-management practices. Several caregivers reported that attendance at diabetes camps and conferences for youth helped alleviate youths’ issues with negative self-image, especially related to the use of diabetes technology. Also, school educators with knowledge about T1D self-management practices helped youth and caregivers feel more empowered.

Reflective listening emerged as a source of support for youth experiencing negative self-image or shame around self-management activities. One Spanish-speaking caregiver addressed their child’s concerns by listening to their child’s preferences about the body location used for glucose monitoring. Another reported that their clinician offered space for reflective listening in clinic appointments: “It’s like making them proud of what they’re learning and the way they’re improving or the way they’re reflecting on their care.” One youth said that when they moved to a new school, it was an opportunity to start over and share with peers about their experiences with T1D.

Meeting with a therapist, shared appointments, and contact with other families of youth with T1D at their diabetes clinics were sources of support for participants in a Spanish speaking focus group. Several caregivers who currently do not have friendships with other caregivers of youth with T1D expressed interest in meeting together as families of youth with T1D.

*A spiritual care pathway encompasses chaplain activities to assess and validate existing coping resources: by discussing coping mechanisms with individuals and facilitating communication about coping between patients or caregivers and the care team*,* chaplains assist individuals with identifying strengths.*

## Discussion

Overall, DREAM focus groups demonstrated that Latinx youth with T1D and their caregivers experience challenges connecting with peers for support about living with diabetes [31]. Findings from our analysis underscore the need for psychosocial support as a part of regular diabetes care and highlight the need for innovations in such interventions [35, 36]. These findings correspond to spiritual care needs, which we defined earlier as the fundamental needs connected to the health and well-being of youth with T1D, such as finding meaning and purpose in living with T1D and feeling a sense of belonging and connection to others, including family, friends, and community. [13].

We found that the problems facing our focus group participants are concerns that professional chaplain clinical activities routinely address [23]. Chaplains hold specific expertise in responding to an individual’s loss of hope, [37] disconnection from community support, [38] difficulty accepting a diagnosis or treatment, [39] grief, [40] and negative self-image [41] and shame. [42] Chaplains also validate individual spiritual strengths and coping resources that support youth’s T1D self-management [23, 43]. Our results included spiritual care pathways for the care of youth with T1D and their caregivers by professional chaplains. These pathways connect to roles for chaplains on the diabetes care team, including performing spiritual assessment, providing a listening ear, offering communication and decision-making support, and serving as a consultant for the T1D team [13, 23].

*Spiritual Assessment*. In our focus groups, participants reported concerns and resources that chaplains routinely assess and help participants recognize in themselves. Chaplains are uniquely trained to perform evidence-based spiritual assessments to identify sources of spiritual strength, signs of spiritual struggle, hopes and fears for youth and families and develop a care plan to address concerns that can be communicated with the full healthcare team [24, 44]. By working with a chaplain’s spiritual assessment, a T1D team can be empowered to understand a youth’s full story and honor the suffering and resilience of the patient in their care [14, 45]. Spiritual assessment can aid the diabetes care team in offering tailored support for youth with T1D and their caregivers. Through the spiritual assessment process, chaplains recognize and help individuals recognize in themselves positive spiritual coping resources (e.g., self-acceptance, supportive communities, prayer, hope). Spiritual assessment can also help the care team understand youth or caregiver beliefs or practices that may contribute to distress (e.g., negative body image, feelings of betrayal by a higher power, fears) [45]. Chaplain involvement in diabetes care could integrate spiritual assessment into the care practices of diabetes care teams and improve the fit between their diabetes care and the whole person. In our sample of Latinx youth and caregivers, spiritual assessment offers an opportunity to directly address the unique role of culture and spiritual needs in their diabetes care.

*A Listening Ear*. Youth and caregivers in our sample named loss of hope, difficulty accepting diagnosis or devices, grief, and negative self-image as issues they face in managing T1D. Several groups discussed the need for people who can understand their experiences to listen to them. Experiences with adversity, such as racial bias, financial hardships, or structural barriers to mental healthcare, intensify the psychological burdens of living with T1D [3, 5]. Also, there is a shortage of mental health professionals in diabetes care, underlining a need for additional support to address unmet psychosocial needs among youth with T1D and their caregivers [35, 46, 47]. Chaplains are trained to offer emotional and spiritual support to patients as well as their families that is not directly focused on behavioral change. Chaplains use a calm, non-judgmental presence to activate a conversational space for reflective listening, allowing individuals to “offload” and name presenting issues [48, 49]. As a member of the interdisciplinary team yet a separate source of support from other diabetes care team members, chaplains could offer youth and caregivers the opportunity to identify their own concerns about diabetes self-management and their daily lives.

Constant worry and diabetes distress are documented challenges for caregivers of youth with T1D [12, 29]. These forms of spiritual struggle bear relevance for chaplain interventions along multiple spiritual care pathways (Table 3). Chaplains explore hope and communicate needs or concerns to clinical team members. Chaplains journey with individuals in the grieving process, helping them to feel comforted and providing resources or grief processing sessions. Chaplains can listen to youth and their caregivers about their life narrative, ask questions to bring forth feelings and meanings of their experiences managing T1D, and demonstrate acceptance of their diagnosis and self-management. Chaplains can also help to preserve dignity and encourage self-respect of youth with T1D and caregivers who experience negative self-image by encouraging self-care practices and offering emotional support. Finally, chaplains can help individuals acknowledge sources of coping support in managing T1D, such as naming how diabetes devices enhance their daily lives, which was reported by our study participants.

*Communication and Decision-making Support.* Our study showed how youth with T1D and caregivers felt disconnected from community support over time in dealing with T1D. Youth reported feeling a need for friendships, but were challenged by the search for other youth with T1D who could relate to them or peers without T1D who were knowledgeable or respectful about their self-management practices. Chaplains routinely help patients identify supportive relationships and work to lessen the feeling of being alone through their interventions. Chaplains companion youth and their families as they face questions about disease management and address related emotional or spiritual values or concerns. Our participants also described ways in which having space with clinicians to reflect on their experiences and connecting with other T1D families helped them to understand and make decisions regarding the use of diabetes technology. When facing decisions about medical care, chaplains can help youth and caregivers understand and process information they have received and advocate for their needs with other members of the clinical team. Also, chaplains seek to align care plans with patient values by asking guided questions about purpose and upon request, perform blessings of medical technology. For instance, when families celebrate the use of a new diabetes device, a chaplain may be consulted to co-design a ritual or blessing of the device with a youth and their family.

*Consultant for the Clinical Team.* Our participants disclosed difficulties coping with T1D, which may be infrequently reported or addressed in the context of routine care appointments. Chaplains can serve as a consultant to the diabetes care team, especially if a youth or caregiver shares experiences of distress in the disclosive space of a chaplain visit. Spiritual struggle is associated with worsening anxiety and depression [10, 11]. A chaplain who is integrated in the diabetes clinic can translate spiritual concerns to other members of the interdisciplinary team and make appropriate referrals for mental health support. Likewise, some of our participants named coping resources or preferences for support that may not be revealed in regular appointments. Chaplains can help shed light on relevant social and cultural clues for improving T1D self-management and device use among youth with T1D and their caregivers [44]. Lastly, a chaplain could facilitate T1D support groups, such as virtual peer groups, or connect youth and caregivers to relevant sources of community support.

While our study results offered relevant findings for management of T1D in youth, limitations exist. First, we were unable to conduct follow-up interviews to generate more detailed responses or solicit feedback on our findings, which may have limited our credibility. Follow-up interviews with youth may have offered opportunity to improve our representation of these participants’ meanings. To address this limitation, in DREAM Phase 2 focus groups, researchers are asking participants about their family’s spiritual coping resources, such as religious/spiritual community. Also, quotations from caregivers were of better length and quality than quotations from youth, resulting in fewer direct quotations from youth than from adults. While the qualitative framework method is suited for multidisciplinary research teams, our analysis team may have been limited due its size (two members) and lack of current care team members. A larger multidisciplinary analysis team may have generated other useful and valid findings from the same focus group data and improved our findings’ credibility.

Prior research suggests that caregivers of youth with T1D use religious coping in their daily lives and this may be more prevalent among Black and Latinx caregivers [2]. Our sample only included Latinx youth with T1D and their caregivers living in California, which limits the transferability of our findings. We did not collect focus group data from a Spanish-speaking focus group of youth with T1D, inhibiting our exploration of the influence of primary language in youth’s experiences. We recruited from three pediatric diabetes clinics and the experiences of those who declined participation may differ from those who decided to participate.

As others have shown, spiritual needs include, but are not limited to, concerns stemming from religious identity or experiences in organized religious communities [24, 26, 28]. The present study describes issues related to managing T1D, which bear relevance to spiritual care interventions and a chaplain’s potential role in the interdisciplinary care team. Nevertheless, another possible limitation of this study is that participants were not asked direct questions about religion or spirituality during the focus groups or in the form of a questionnaire. Lessons from this study have informed our team’s next research study, which explores caregiver experiences using religion or spirituality to cope with their child’s T1D through in-depth interviews and questionnaires to assess spiritual well-being and identify perceptions about professional chaplaincy.

## Conclusions

Latinx youth with T1D and their caregivers described life experiences and emotions related to managing T1D, which we viewed through the lens of spiritual care. To our knowledge, our research is the first to identify specific unmet needs in T1D care and focus on the benefits of spiritual care interventions and chaplains as part of the multidisciplinary team [29]. The Association of Diabetes Care and Education Specialists framework (ADCES7) for self-care behaviors includes “participating in faith-based activities” as a suggested form of “healthy coping.” [50]. Yet, youth with T1D and their caregivers possibly use various spiritual coping resources that are untapped by healthcare professionals [12]. Spiritual struggle related to managing T1D (e.g., isolation from others with T1D, negative self-image, grief) warrants specialized support [51, 52]. Our results highlight how an integrated chaplain in diabetes care delivery may enhance the utilization and efficacy of support and management services for youth living with diabetes by providing spiritual assessment of distress and collaborating with other diabetes team members in delivering support.

Spirituality is as important and complex a characteristic as race, culture, age, or gender, but it is not frequently included in psychosocial and behavioral research [28, 53]. The present study challenges the current clinical practice and research paradigm by highlighting the need for spiritual care support among Latinx youth with T1D and their caregivers. Furthermore, it also identifies opportunities for chaplains to be a part of clinical care to assess and address any spiritual needs or challenges and provide additional support. This is especially critical for historically marginalized communities, where addressing unmet needs has the potential to improve health outcomes. More research is needed to identify the perceptions and preferences for chaplain integration in diabetes care among youth with T1D, caregivers, andclinicians.

## Data Availability

The datasets supporting the conclusions of this article are not publicly available due HIPAA, Data User Agreements, and the consent process. Data are only shared with the research team and the funding agency.

## Abbreviations

T1D: Type 1 Diabetes
DREAM: Device use Reimagined through Education And Mentorship
CHLA: Children’s Hospital Los Angeles
UCD: University of California, Davis
UCSF: University of California, San Francisco
HbA1c: Hemoglobin A1c
CGM: Continuous Glucose Monitoring

## References

[1] National Diabetes Statistics Report | Diabetes | CDC. Accessed March 26. 2026. https://www.cdc.gov/diabetes/php/data-research/index.html

[2] Lawrence JM, et al. Trends in Prevalence of Type 1 and Type 2 Diabetes in Children and Adolescents in the US, 2001–2017. JAMA. 2021;326(8):717–27. 34427600 10.1001/jama.2021.11165 PMC8385600

[3] Agarwal S, Kanapka LG, Raymond JK, et al. Racial-Ethnic Inequity in Young Adults With Type 1 Diabetes. J Clin Endocrinol Metabolism. 2020;105(8):e2960–9. 10.1210/clinem/dgaa236. 10.1210/clinem/dgaa236 PMC7457963 32382736

[4] Ebekozien O, Mungmode A, Sanchez J, et al. Longitudinal Trends in Glycemic Outcomes and Technology Use for Over 48,000 People with Type 1 Diabetes (2016–2022) from the T1D Exchange Quality Improvement Collaborative. Diabetes Technol Ther. 2023;25(11):765–73. 10.1089/dia.2023.0320. 37768677 10.1089/dia.2023.0320

[5] Roberts A, Corathers S, Rapaport R, et al. Depression Rates in Youth With Type 1 Diabetes During the COVID-19 Pandemic: Data From the T1D Exchange Quality Improvement Collaborative. Clin Diabetes. 2024;42(4):532–9. 10.2337/cd24-0004. 39429445 10.2337/cd24-0004 PMC11486847

[6] Willi SM, Miller KM, DiMeglio LA, et al. Racial-ethnic disparities in management and outcomes among children with type 1 diabetes. Pediatrics. 2015;135(3):424–34. 10.1542/peds.2014-1774. 25687140 10.1542/peds.2014-1774 PMC4533245

[7] Tsai D, Flores Garcia J, Fogel JL, Wee CP, Reid MW, Raymond JK. Diabetes Technology Experiences. 10.1177/19322968211029260 PMC9264427 34225480

[8] Among Latinx and Non-Latinx Youth with Type 1 Diabetes. J Diabetes Sci Technol. SAGE Publications Inc. 2021;19322968211029260.

[9] da Silva N, Dillon FR, Verdejo TR, Sanchez M, De La Rosa M. Acculturative Stress, Psychological Distress, and Religious Coping Among Latina Young Adult Immigrants. Couns Psychol. 2017;45(2):213–36. 10.1177/0011000017692111. 29033462 10.1177/0011000017692111 PMC5636182

[10] Alvarenga W, de Machado A, Leite JR. Spiritual Needs of Brazilian Children and Adolescents with Chronic Illnesses: A Thematic Analysis: Journal of Pediatric Nursing. J PEDIATR NURS. 2021;60:e39–45. 10.1016/j.pedn.2021.02.020. 33648836 10.1016/j.pedn.2021.02.020

[11] da Silva AP, Araujo ACMC, Mesquita IMR, et al. Religious/spiritual coping, symptoms of depression, stress, and anxiety in caregivers of children and adolescents with type 1 diabetes. Fam Pract. 2022;39(6):1017–23. 10.1093/fampra/cmac032. 35477768 10.1093/fampra/cmac032

[12] Fitchett G, Murphy PE, Kim J, Gibbons JL, Cameron JR, Davis JA. Religious Struggle: Prevalence, Correlates and Mental Health Risks in Diabetic, Congestive Heart Failure, and Oncology Patients. Int J Psychiatry Med. 2004;34(2):179–96. 10.2190/ucj9-dp4m-9c0x-835m. 15387401 10.2190/UCJ9-DP4M-9C0X-835M

[13] Joiner KL, DeJonckheere M, Whittemore R, Grey M. Perceptions and experiences of living with type 1 diabetes among Latino adolescents and parents with limited English proficiency. Res Nurs Health. 2020;43(3):263–73. 10.1002/nur.22019. 32281136 10.1002/nur.22019

[14] Kestenbaum A, Shields M, James J, et al. What Impact Do Chaplains Have? A Pilot Study of Spiritual AIM for Advanced Cancer Patients in Outpatient Palliative Care. J Pain Symptom Manage. 2017;54(5):707–14. 10.1016/j.jpainsymman.2017.07.027. 28736103 10.1016/j.jpainsymman.2017.07.027 PMC5650916

[15] Puchalski C et al. Foreword. In: Bull AW, Zollfrank A, Hildebrand A, editors. Spiritual Care in Practice: Case Studies in Healthcare Chaplaincy. Jessica Kingsley; 2015:9-11.

[16] Jeanne Wirpsa M, Emily Johnson R, Bieler J, et al. Interprofessional Models for Shared Decision Making: The Role of the Health Care Chaplain. J Health Care Chaplain. 2019;25(1):20–44. 10.1080/08854726.2018.1501131. 30321119 10.1080/08854726.2018.1501131

[17] Rajaee G, Patel MR. Preferences for healthcare chaplaincy services among U.S. adults: differences by inpatient and outpatient settings. J Health Care Chaplain. 2023;29(2):161–75. 10.1080/08854726.2022.2064125. 35446754 10.1080/08854726.2022.2064125

[18] Handzo G, Hughes B, Bowden J, et al. Chaplaincy in the outpatient setting-getting from here to there. J Health Care Chaplain. 2022;28(2):194–207. 10.1080/08854726.2020.1818359. 32981466 10.1080/08854726.2020.1818359

[19] Sprik PJ, Janssen Keenan A, Boselli D, Grossoehme DH. Chaplains and telechaplaincy: best practices, strengths, weaknesses—a national study. J Health Care Chaplain. 2023;29(1):41–63. 10.1080/08854726.2022.2026103. 35067213 10.1080/08854726.2022.2026103

[20] Bezabih A, Nourriz S, Snider AM, Rauenzahn R, Handzo G, Smith CE. Meeting Patients Where They’re At: Toward the Expansion of Chaplaincy Care into Online Spiritual Care Communities. Proc ACM Hum-Comput Interact. 2025;9(7):1–38. 10.1145/3757492.40909183

[21] Butler AM, Hilliard ME, Titus C, et al. Barriers and Facilitators to Involvement in Children’s Diabetes Management Among Minority Parents. J Pediatr Psychol. 2020;45(8):946–56. 10.1093/jpepsy/jsz103. 31995219 10.1093/jpepsy/jsz103 PMC7828466

[22] Hsin O, La Greca AM, Valenzuela J, Moine CT, Delamater A. Adherence and Glycemic Control among Hispanic Youth with Type 1 Diabetes: Role of Family Involvement and Acculturation. J Pediatr Psychol. 2010;35(2):156–66. 10.1093/jpepsy/jsp045. 19491214 10.1093/jpepsy/jsp045 PMC2821010

[23] Hunter-Hernández M, Costas-Muñíz R, Gany F. Missed Opportunity: Spirituality as a Bridge to Resilience in Latinos with Cancer. Journal of Religion and Health [Internet]. 2015 [cited 2024 Mar 8];54(6):2367–75. Available from: https://www.jstor.org/stable/24735969 10.1007/s10943-015-0020-y PMC4961247 25711211

[24] Massey K, Barnes MJ, Villines D, Goldstein JD, Pierson ALH, Scherer C, et al. What do I do? Developing a taxonomy of chaplaincy activities and interventions for spiritual care in intensive care unit palliative care. BMC Palliat Care. 2015;14(1):10. 10.1186/s12904-015-0008-0. 25878558 10.1186/s12904-015-0008-0 PMC4397872

[25] Fitchett G. Assessing Spiritual Needs. Academic Renewal Press, 2002.

[26] Henderson KK, Oliver JP, Hemming P. Patient Religiosity and Desire for Chaplain Services in an Outpatient Primary Care Clinic. J Pastoral Care Counsel. 2023;77(2):81–91. 10.1177/15423050221147901. 36660791 10.1177/15423050221147901

[27] Trevino KM, Pargament KI, Krause N, Ironson G, Hill P. Stressful events and religious/spiritual struggle: Moderating effects of the general orienting system. Psychol Relig Spiritual. 2019;11(3):214–24. 10.1037/rel0000149.supp. Located at: 2017-53719-001.

[28] Currier JM, Foster JD, Witvliet C, vanOyen, Abernethy AD, Root Luna LM, Schnitker SA, et al. Spiritual struggles and mental health outcomes in a spiritually integrated inpatient program. J Affect Disord. 2019;249:127–35. 10.1016/j.jad.2019.02.012. 30772739 10.1016/j.jad.2019.02.012

[29] White K, Jennings J, ’Aime C, Karimi S, Johnson CE, Fitchett G. Examining Factors Associated with Utilization of Chaplains in the Acute Care Setting. J Relig Health. 2022;61(2):1095–119. 10.1007/s10943-021-01460-x. 34797457 10.1007/s10943-021-01460-x

[30] Baudino MN, Garcia Perez S, O’Donnell MB, Duran B, DeSalvo DJ, Malik F, et al. Faith, Family, and Friends: Pandemic-Related Coping in Parents of Adolescents With Type 1 Diabetes. Clin Pract Pediatr Psychol. 2024;12(4):457–67. 10.1037/cpp0000528. 40726631 10.1037/cpp0000528 PMC12290988

[31] Pargament KI, Smith BW, Koenig HG, Perez L. Patterns of Positive and Negative Religious Coping with Major Life Stressors. J Sci Study Relig. 1998;37(4):710–24. 10.2307/1388152.

[32] Wong JC, et al. Connecting and personalizing resources to support diabetes technology use: Perspectives of Latinx families with type 1 diabetes. Horm Res Paediatr. 2024;97:228–9.

[33] Gale NK, Heath G, Cameron E, Rashid S, Redwood S. Using the framework method for the analysis of qualitative data in multi-disciplinary health research. BMC Med Res Methodol. 2013;13(1):117. 10.1186/1471-2288-13-117. 24047204 10.1186/1471-2288-13-117 PMC3848812

[34] Dedoose. Dedoose Version 10.0.59 [Internet]. Los Angeles (CA): SocioCultural Research Consultants, LLC; 2026. Available from: https://www.dedoose.com

[35] Puchalski C, Ferrell B, Virani R, Otis-Green S, Baird P, Bull J et al. Improving the quality of spiritual care as a dimension of palliative care: the report of the Consensus Conference. J Palliat Med. 2009;12(10):885–904. 10.1089/jpm.2009.0142 PubMed PMID: 19807235. 10.1089/jpm.2009.0142 19807235

[36] Dimentstein K, Greenberg BA, Valenzuela JM. Involvement of Racially and Ethnically Minoritized Youths in Behavioral Type 1 Diabetes Interventions: A Systematic Review. J Pediatr Psychol. 2023;48(5):428–47. 10.1093/jpepsy/jsad018. 37016949 10.1093/jpepsy/jsad018

[37] Carlsund Å, Olsson S, Hörnsten Å. Stress and Burden Experienced by Parents of Children with Type 1 Diabetes—A Qualitative Content Analysis Interview Study. Children. 2025;12(8):984. 10.3390/children12080984. 40868436 10.3390/children12080984 PMC12384735

[38] Binaei N, Moeini M, Sadeghi M, Najafi M, Mohagheghian Z. Effects of hope promoting interventions based on religious beliefs on quality of life of patients with congestive heart failure and their families. Iran J Nurs Midwifery Res. 2016;21(1):77–83. 10.4103/1735-9066.174755 . PubMed PMID: 26985226; PubMed Central PMCID: PMC4776564. 26985226 10.4103/1735-9066.174755 PMC4776564

[39] White KB, Galchutt P, Collier K, Szilagyi C, Fitchett G. Spiritual Care and Community Wellness: A Mixed-Methods Approach to Explore Chaplains’ Integration in Community Health Initiatives. Delcea C, editor. Health & Social Care in the Community. 2025;2025(1):6128995. 10.1155/hsc/6128995

[40] Michelson KN, Arenson M, Charleston E, Clayman ML, Brazg T, Rychlik K, et al. Parental Views of Social Worker and Chaplain Involvement in Care and Decision Making for Critically Ill Children with Cancer. Child (Basel). 2022;9(9):1287. 10.3390/children9091287 . PubMed PMID: 36138595; PubMed Central PMCID: PMC9497868. 10.3390/children9091287 PMC9497868 36138595

[41] Jeuland J, Fitchett G, Schulman-Green D, Kapo J. Chaplains Working in Palliative Care: Who They Are and What They Do. J Palliat Med. 2017;20(5):502–8. 10.1089/jpm.2016.0308. PubMed PMID: 28146647. 28146647 10.1089/jpm.2016.0308

[42] Crandall RJ. The Psychosocial Aspect of Burn Patient Recovery as it Informs Chaplain Interventions. J Pastoral Care Counsel. 2020;74(3):175–81. 10.1177/1542305020949830. 32967550 10.1177/1542305020949830

[43] Sprik PJ, Walsh K, Boselli DM, Meadors P. Using patient-reported religious/spiritual concerns to identify patients who accept chaplain interventions in an outpatient oncology setting. Support Care Cancer. 2019;27(5):1861–9. 10.1007/s00520-018-4447-z . PubMed PMID: 30187222. 30187222 10.1007/s00520-018-4447-z

[44] Haufe M, Leget C, Potma M, Teunissen S. How can existential or spiritual strengths be fostered in palliative care? An interpretative synthesis of recent literature. BMJ Support Palliat Care. 2024;14(3):279–89. 10.1136/bmjspcare-2020-002379. 32928785 10.1136/bmjspcare-2020-002379 PMC11347204

[45] Association of Professional Chaplains. (2015) Standards of Practice for Professional Chaplains. Accessed on 3/20/2026 at www.professionalchaplains.org/content.asp?pl=198&sl=198&contentid=200

[46] Robert R, Stavinoha P, Jones BL, Robinson J, Larson K, Hicklen R, et al. Spiritual assessment and spiritual care offerings as a standard of care in pediatric oncology: A recommendation informed by a systematic review of the literature. Pediatr Blood Cancer. 2019;66(9):e27764. 10.1002/pbc.27764. 31033210 10.1002/pbc.27764

[47] Versloot J, Ali A, Minotti SC, Ma J, Sandercock J, Marcinow M, et al. All together: Integrated care for youth with type 1 diabetes. Pediatr Diabetes. 2021;22(6):889–99. 10.1111/pedi.13242. 34173306 10.1111/pedi.13242 PMC9290723

[48] Price J, Lewis AM, Pierce JS, Enlow PT, Okonak K, Kazak AE. Psychosocial Staffing and Implementation of the International Society for Pediatric and Adolescent Diabetes Psychological Care Guidelines in U.S. Pediatric Diabetes Clinics. Diabetes Spectr. 2023;36(3):219–27. 10.2337/ds22-0047. 37583560 10.2337/ds22-0047 PMC10425227

[49] McSherry W, Boughey A, Kevern P. Chaplains for Wellbeing in Primary Care: A Qualitative Investigation of Their Perceived Impact for Patients’ Health and Wellbeing. J Health Care Chaplain. 2016;22(4):151–70. 10.1080/08854726.2016.1184504. 27254073 10.1080/08854726.2016.1184504

[50] Damen A, Schuhmann C, Rosie XJS, van Zundert M, Jacobs G, Muthert H, et al. The Contribution of Chaplaincy to Primary and Community Care: A Semi-Structured Interview Study With Clients. J Prim Care Community Health. 2025;16:21501319251357528. 10.1177/21501319251357528 . PubMed PMID: 40673499; PubMed Central PMCID: PMC12276415. 40673499 10.1177/21501319251357528 PMC12276415

[51] Association of Diabetes Care & Education Specialists. ADCES7 Self-Care Behaviors. https://www.adces.org/diabetes-education-dsmes/adces7-self-care-behaviors. Accessed 1 May 2026.

[52] de Lucca M, Visser M, Andre T, Leturia S, Nascimento L, Barber R. Mapping Barriers and Interventions to Diabetes Self-Management in Latino Youth: A Scoping Review. Children. 2025;12(7):882. 10.3390/children12070882. 40723075 10.3390/children12070882 PMC12293598

[53] Park CL. Estimated longevity and changes in spirituality in the context of advanced congestive heart failure. Pall Supp Care. 2008;6(1):3–11. 10.1017/S1478951508000023. 10.1017/S1478951508000023 18282339

